# Tumors Involving the Infratemporal Fossa: A Systematic Review of Clinical Characteristics and Treatment Outcomes

**DOI:** 10.3390/cancers14215420

**Published:** 2022-11-03

**Authors:** Othman Bin-Alamer, Lokeshwar S. Bhenderu, Paolo Palmisciano, Kishore Balasubramanian, Prashant Upadhyay, Gianluca Ferini, Anna Viola, Valentina Zagardo, Kenny Yu, Aaron A. Cohen-Gadol, Tarek Y. El Ahmadieh, Ali S. Haider

**Affiliations:** 1 Department of Neurosurgery, University of Pittsburgh Medical Center, Pittsburgh, PA 15213, USA; 2 Department of Neuroscience and Experimental Therapeutics, Texas A&M University Health Science Center, Texas A&M University, Bryan, TX 77807, USA; 3 Department of Neurosurgery, University of Cincinnati College of Medicine, Cincinnati, OH 45267, USA; 4 Faculty of Medicine, Government Medical College Jalaun, Orai 285001, Uttar Pradesh, India; 5 Department of Radiation Oncology, REM Radioterapia srl, 95125 Viagrande, Italy; 6 Department of Neurosurgery, Memorial Sloan Kettering Cancer Center, New York, NY 10065, USA; 7 Department of Neurological Surgery, Indiana University School of Medicine, Indianapolis, IN 46202, USA; 8 Department of Neurosurgery, Loma Linda University, Loma Linda, CA 92350, USA; 9 Department of Neurosurgery, The University of Texas M.D. Anderson Cancer Center, Houston, TX 77030, USA

**Keywords:** infratemporal fossa, skull base, meningiomas, schwannomas, transcranial surgery, endoscopic endonasal surgery

## Abstract

**Simple Summary:**

Located in the lateral facial region, the infratemporal fossa (ITF) is the primary site for tumors of various etiologies and comprise 0.5% of all head and neck cancers. Due to the anatomical relationship of ITF tumors with different cranial nerves and neurovascular structures, clinical presentations vary among patients. Our study aims to review the literature on the various tumors that present in this region, their reported treatment strategies, and patient outcomes. We found that trigeminal schwannomas and meningiomas are the most common tumors. In terms of management, nost patients had transcranial surgery, and three-quarters had a gross-total resection.

**Abstract:**

Background: Infratemporal fossa (ITF) tumors represent various pathologies and are seldom described in the literature, reflecting their rarity. Here we review the literature on tumors invading ITF and describe patient characteristics, treatment strategies, and clinical outcomes. Methods: Relevant articles were retrieved from PubMed, Scopus, and Cochrane. A systematic review and meta-analysis were conducted on the clinical presentation, treatment protocols, and clinical outcomes. Result: A total of 27 articles containing 106 patients with ITF tumors (median tumor size: 24.3 cm^3^ [interquartile range, 15.2–42 cm^3^]) were included (median age: 46 years [interquartile range, 32–55 years]; 59.4% were males]). Of the confirmed tumor pathology data, schwannomas (*n* = 24; 26.1%) and meningiomas (*n* = 13; 14.1%) were the most common tumors. Facial hypoesthesia (*n* = 22; 18.5%), auricular/preauricular pain (*n* = 20; 16.8%), and headaches (*n* = 11; 9.2%) were the most common presenting symptoms. Of patients who had surgical resection (*n* = 97; 95.1%), 70 (73.7%) had transcranial surgery (TCS) and 25 (26.3%) had endoscopic endonasal surgery (EES). Among available details on the extent of resection (*n* = 84), gross-total resection (GTR) was achieved in 62 (73.8%), and 5 (6.0%) had biopsy only. Thirty-five (33.0%) patients had postoperative complications. Among cases with available data on reconstruction techniques (*n* = 8), four (50%) had adipofascial antero-lateral thigh flap, three (37.5%) had latissimus dorsi free flap, and one (12.5%) had antero-lateral thigh flap. Fourteen (13.2%) patients had adjuvant chemotherapy, and sixteen (15.1%) had adjuvant radiotherapy. During a median follow-up time of 28 months (IQR, 12.25–45.75 months), 15 (14.2%) patients had recurrences, and 18 (17.0%) patients died. The median overall survival (OS) time was 36 months (95% confidence interval: 29–41 months), and the 5-year progression-free survival (PFS) rate was 61%. Conclusion: Various tumor types with different biological characteristics invade the ITF. The present study describes patient demographics, clinical presentation, management, and outcomes. Depending on the tumor type and patient condition, patient-tailored management is recommended to optimize treatment outcomes.

## 1. Introduction

Although approximately 80% are benign, infratemporal fossa (ITF) tumors are highly heterogenous. They include various tumor subtypes such as schwannomas, meningiomas, and sarcomas [1,2,3,4,5]. Due to the proximity of ITF tumors to different cranial nerves and neurovascular structures, ITF tumors also pose a surgical challenge and have highly variable clinical symptoms at presentation [1,2,3,4,5].

Although it varies based on the specific tumor type, the management paradigm of ITF tumors relies mainly on a combination of surgical resection, chemotherapy and/or radiotherapy [1,2,3,5]. Historically, ITF tumors were treated invasively with disfiguring surgical resections which carried a high risk of complications and high morbidity and mortality rates [1,6,7]. However, the past decade has provided major advances in skull base microsurgery techniques, ranging from transcranial (TCS) to endoscopic endonasal surgery (EES) [8,9]. EES allows for a more expansive view of the skull base and its feasibility has greatly improved since early technical reports. This has led to an increased use of EES in ITF tumor management [10].

Despite the clinical importance of ITF tumors, the literature relies on case reports and single-center experiences [1,2,3,5]. In the present study, we reviewed the current literature, summarized the available data on ITF tumors, and described the current management protocols and their clinical outcomes.

## 2. Materials and Methods

### 2.1. Literature Search

A systematic review, registered to PROSPERO (ID: CRD42022362805), was conducted per the Preferred Reporting Items for Systematic Reviews and Meta-Analyses (PRISMA) guidelines [11]. PubMed, Scopus, and Cochrane databases were searched from inception to June 2021. A medical subject headings (MeSH) term and keyword search of each database was conducted using the Boolean operators OR and AND. Terms used were as follows: “infratemporal fossa” AND “carcinoma OR cancer OR tumor OR malignancy.” Identified papers were uploaded into Mendeley (Versioin 2.80.1, Mendeley Ltd., 27 October 2022, London, England), and duplicates were eliminated.

### 2.2. Study Selection

Inclusion and exclusion criteria were predetermined. Studies were included if they: (1) involved patients with skull base tumors located within the ITF, (2) reported data on clinical features, treatment protocols, and outcomes; (3) were written in the English language. Studies were excluded if they: (1) were literature reviews, case reports, technical notes, abstracts, or autopsy reports; (2) did not clearly differentiate data of patients with ITF tumors from data of patients with tumors in different anatomical locations; or (3) lacked treatment and outcome data.

Two authors (P.U. and O.B.-A.) independently assessed the titles and abstracts of all extracted papers based on the inclusion and exclusion criteria. Studies that met inclusion criteria were then further evaluated independently with full text review by the same two authors, and disagreements were resolved via a third author (A.S.H.). References of the included articles were also screened to retrieve additional relevant articles.

### 2.3. Data Extraction

Data from included studies were extracted by one author (L.S.B.) and confirmed independently by two authors (O.B.-A. and P.P.) to ensure accuracy. Extraction variables included: (1) author’s name, (2) date of publication, (3) level of evidence, (4) sample size, (5) sex, (6) presenting symptoms, (7) histological and radiological features, (8) management course and treatment modalities used (radiotherapy, chemotherapy, surgical approach), (9) recurrence, (10) survival outcomes. Missing data are either not reported by the original article or reported indistinctively from other data. Tumor volumes were reported as they were reported in the included articles. If the volume was not reported, radiological dimensions were used instead, if available.

### 2.4. Data Synthesis and Quality Assessment

The primary outcomes of interest were the characteristics of ITF tumors, the overall survival (OS) and progression-free survival (PFS) of patients, and additional predictive survival factors. The level of evidence of each article was evaluated following the 2011 Oxford Centre for Evidence-Based Medicine guidelines, and all articles were categorized as level IV evidence [12]. The risk of bias was independently assessed for each article by two authors (O.B.-A. and P.P.) using the Joanna Briggs Institute checklists [13]. Risk-of-bias assessment resulted in a low risk of bias in all included papers (Supplementary Table S1).

### 2.5. Statistical Analysis

R (Version 4.2.2, RStudio, Inc., R Foundation for Statistical Computing, Vienna, Austria, http://www.R-project.org/, accessed on 31 October 2022) was used for all statistical analyses. Continuous variables were summarized as median and interquartile ranges (IQR), while categorical variables were summarized as frequencies and percentages. The survival data were reported as median months (95% confidence interval [CI]). Not applicable (NA) in the CI limits indicates infinity due to the skewness of the data. Chi-square analyses were used to test significant differences between categorical variables. Using the R package ‘survival’, OS and PFS were calculated using Kaplan–Meier curves. The Cox proportional hazard model was used for the univariable and multivariable analyses to evaluate factors potentially affecting survival. For testing the proportional hazards assumption, Schoenfeld’s global test was used to estimate time-varying covariance. The schwannoma tumor type violated the proportional hazards assumption in the OS Cox proportional hazard model and was employed as a stratifying factor. Logistic regression analysis for complications was conducted by testing multiple patient and treatment factors. Continuous variables were categorized based on the most significant point based on Log rank testing. A two-tailed *p*-value < 0.05 was deemed to be significant for all analyses.

## 3. Results

### 3.1. Study Selection

**Figure 1:**
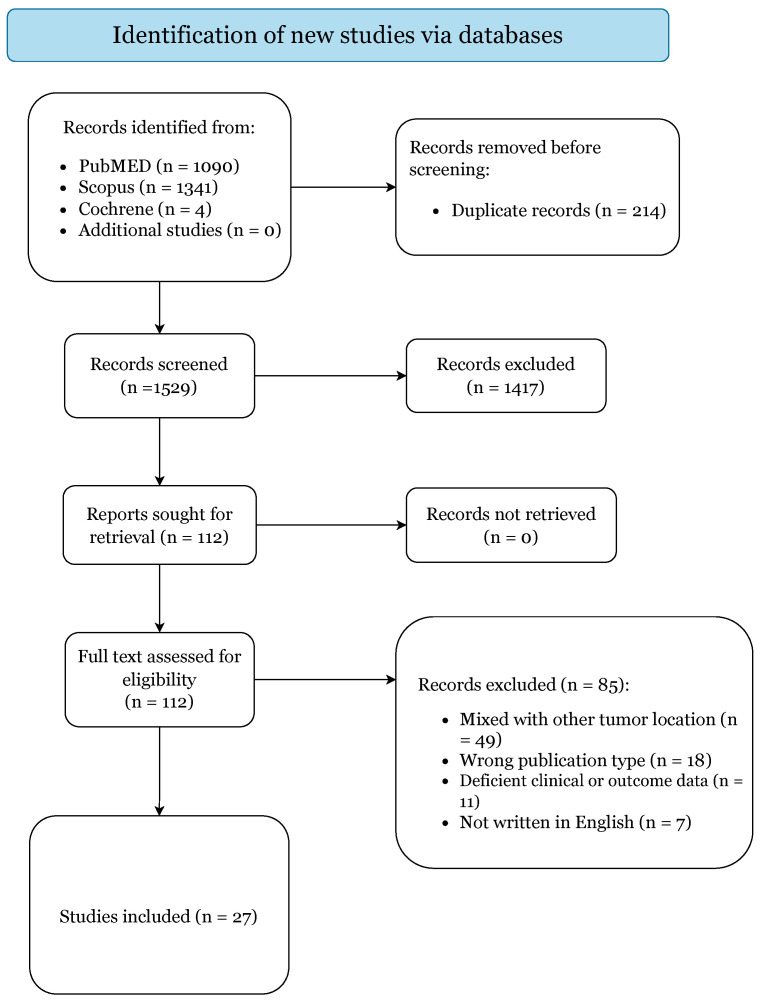
PRISMA 2020 Flow-Diagram.

The initial literature search yielded 2435 citations (Figure 1).

After elimination of duplicates, there were 1529 articles. A total of 1417 studies were excluded based on title and abstract screening. Of the 112 papers selected for retrieval, 85 articles failed to meet our inclusion criteria and were subsequently excluded. Thus, 27 articles were included in this systematic review based upon the prespecified criteria (Supplementary Table S2) [1,2,3,6,7,14,15,16,17,18,19,20,21,22,23,24,25,26,27,28,29,30,31,32,33,34,35].

### 3.2. Demographics and Clinical Features

The present study included one hundred six patients with ITF tumors (median tumor size: 24.3 cm^3^ [IQR, 15.2–42 cm^3^]). The cohort’s median age was 46 years (IQR, 32–55 years), and males constituted 59.4% (*n* = 63) of the demographics (Table 1).

**Figure 2:**
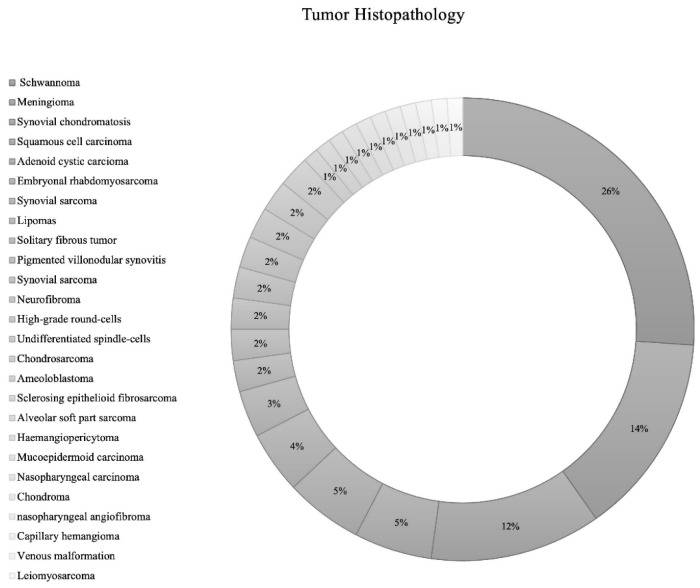
Pie chart of tumor histopathology.

The most common structures invaded by ITF tumors were pterygopalatine fossa (*n* = 29; 26.1%), temporomandibular joint (*n* = 14; 12.6%), and the orbit (*n* = 14; 12.6%) (Table 1). While the included IFT tumors had various etiologies., the most common tumor types were schwannoma (*n* = 24; 26.1% [Trigeminal schwannoma *n* = 19; malignant schwannoma *n* = 1; facial schwannoma *n* = 1; unspecified cranial nerve schwannoma *n* = 3]), followed by meningioma (*n* = 13; 14.1% [grade 1 = 12; grade 2 = 1]), synovial chondromatosis (*n* = 11; 12.0%), squamous cell carcinoma (*n* = 5; 5.4%), and adenoid cystic carcinoma (*n* = 5; 5.4%) (Table 1; Figure 2).

The most common presenting symptoms included facial hypoesthesia (*n* = 22; 18.5%), auricular/preauricular pain (*n* = 20; 16.8%), headaches (*n* = 11; 9.2%), jaw deviation (*n* = 11; 9.2%), hearing loss (*n* = 9; 7.6%), and facial pain (*n* = 8; 6.7%). The trigeminal nerve was the most commonly impacted cranial nerve (*n* = 7; 46.7%), followed by the abducent (*n* = 3; 20.0%) and oculomotor (*n* = 2; 13.3%) cranial nerves (Table 1).

### 3.3. Management Paradigm and Postoperative Complications

Ninety-seven (95.1%) patients had surgical resections. Of these patients, 70 (73.7%) had TCS, and 25 (26.3%) had EES. The surgical method was not specified in two cases. Among cases with details on surgical approach, the most employed surgical approach was mandibulotomy (*n* = 16; 17.8%), followed by condylotomy with posterior disc attachment release (*n* = 14; 15.6%), unspecified endoscopic endonasal approach (*n* = 13; 14.4%), and middle fossa/zygomatic approach (*n* = 9; 10.0%). Of the cases that specified the extent of resection (*n* = 84), 62 (73.8%) patients had gross-total resection (GTR), 17 (20.2%) had subtotal resection (STR), and 5 (6.0%) had biopsy only. Among the various tumors, EES was employed in 19 (86%) trigeminal schwannomas, 2 (16.7%) benign meningiomas, 1 (33%) squamous cell carcinoma, and 1 (100%) atypical meningioma fibrous meningioma (Table 2; *p* < 0.01). The rest of the tumors had TCS.

A total of 35 (33.0%) patients had surgical complications. The most common complications were lingual nerve complication (*n* = 8; 22.9%), inferior alveolar nerve complication (*n* = 7; 20.0%), and facial paresis (*n* = 7; 20.0%; Table 1). Complication rates were significantly higher among patients that had TCS (47.1%) when compared to EES (8.0%; *p* = 0.01; Table 2). Complication rates differed by surgical approach (Table 2; *p* < 0.01), and the highest complication rates were associated with the parotidectomy incision approach (100%), the transzygomatic arch approach (100%), and the latero-facial approach (100%).

Reconstruction techniques and material were reported in eight patients: four (50%) had adipofascial antero-lateral thigh flap, three (37.5%) had latissimus dorsi free flap, and one (12.5%) had antero-lateral thigh flap (Table 1).

Fourteen (13.2%) patients had adjuvant chemotherapy. One (7.1%) patient had Methotrexate, one (7.1%) had Cisplatin, one (7.1%) had Ifosfamide, and one (7.1%) had Doxorubicin. However, the chemotherapeutic agent was unknown in 10 (71.4%) patients.

Sixteen (15.1%) patients had adjuvant radiotherapy. Three (18.8%) had external beam radiotherapy, two (12.5%) had Gamma Knife radiosurgery, and one (6.3%) had proton beam therapy. However, radiotherapy modality was not specified in 10 (62.5%) patients (Table 1).

### 3.4. Patient Clinical and Survival Outcomes

**Figure 3:**
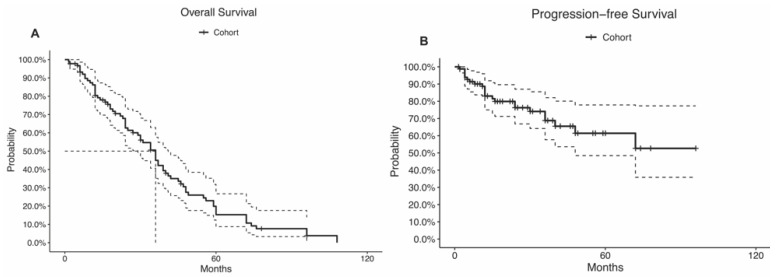
Kaplan–Meier curves of the (**A**) overall survival and (**B**) progression-free survival. The curve doted lines represent the 95% confidence intervals. The doted straight lines represent the. median.

During a median follow-up time of 28 months (IQR, 12.25–45.75 months), 15 (14.2%) patients had a tumor recurrence, and 18 (17.0%) patients died. The recurrence rate was highest among alveolar soft part sarcoma (*n* = 1; 100%), sclerosing epithelioid fibrosarcoma (*n* = 1; 100%), atypical meningioma (*n* = 1; 100%), capillary hemangioma (*n* = 1; 100%), and synovial sarcoma (*n* = 3; 60%; *p* = 0.04; Table 2). The mortality rate was highest among neurofibroma (*n* = 2; 100%), undifferentiated spindle cells (*n* = 2; 100%), alveolar soft part sarcoma (*n* = 1; 100%), and sclerosing epithelioid fibrosarcoma (*n* = 1; 100%; *p* < 0.01; Table 2). The 5-year OS rate was 20%, and the median OS time was 36 months (95% CI: 29–41 months; Table 1; Figure 3A). The 5-year PFS rate was 61%, and the median PFS time was not reached (95% CI: 48.0-NA; Table 1; Figure 3B). Disparity in the survival results was because the progression status was not reported in many patients and was considered censored.

Multivariable analyses of OS (Table 3) and PFS (Table 4) Cox proportional hazards did not result in any significant predictors.

Similarly, the multivariable logistic regression analyses did not detect any significant correlation between postoperative complications and any of the patient or treatment factors (Table 5).

## 4. Discussion

Our systematic review found high rates of schwannoma and meningioma among tumors involving the ITF. Most of these tumors were resected using transcranial surgery (73.7%), and gross-total resection was achieved in 20.2% of cases. The median OS time was 36 months (95% CI: 29–41 months), and the 5-year PFS rate was 61%. The multivariable analysis did not detect any significant predictors of OS, PFS, or complication rates.

### 4.1. Patient Clinical Characteristics

In line with other skull base neoplastic lesions, the median age was 46 years and 59.4% of the patients were males [36,37,38]. The pterygopalatine fossa, temporomandibular joint, and the orbit were the most invaded structures [39,40]. The most common tumor types in our study were meningiomas and schwannomas. This is more a reflection of the general prevalence of these tumor types, as meningiomas and schwannomas are among the most common primary intracranial tumors [41,42,43].

The clinical presentation of ITF tumors in our study mainly included facial hypoesthesia, auricular/preauricular pain, headaches, and jaw deviation. Clinical symptoms were concordant with the anatomical location affecting the surrounding craniofacial structures [44,45].

### 4.2. Management and Survival Outcomes

In our study, 73.7% of the cohort had a transcranial approach. However, our review encompasses data in studies published before the EES era. While different transcranial approaches have been used since then, EES has been increasingly adopted in the skull base literature due to its low postoperative complications and shorter preparative stay [46,47,48]. Several authors have further advanced the techniques and proposed alternative approaches which has led to maximized exposure of the posterolateral wall of the maxillary sinus [1,39,40]. We found that the EES was more frequently implemented for benign tumors, such as trigeminal schwannomas and meningiomas, that are accessible by endoscopic endonasal access, whereas TCS was mainly employed for malignant tumors with extracranial extension. This trend was corroborated in other reports, where tumors resected using EES appeared to be largely benign and anatomically located within the field of endoscopic endonasal access [49,50]. The data represents the clinical paradigm of managing primary malignant disease with radical resections, despite correspondingly higher comorbidity, whereas a partial resection could still yield good long-term results in the setting of benign tumors. Although postoperative complications were significantly more common among patients who had TCS than EES, our logistic regression analysis did not find any significant predictors of complications. However, we attribute the absence of significance to the small sample size among which complication details were reported. An increasing body of the literature has shown that EES has offered a minimally invasive option while achieving comparative extents of resection for different anterior and middle skull base lesions; although conclusive results are yet to be published [47,51,52]. In a comparative analysis, Bander et al. [53] compared EES and TCS for tuberculum sellae and planum sphenoidale meningiomas. They found a higher rate of visual symptoms improvement (EES: 93% vs. TCS: 56%; *p* = 0.049) and a lower rate of visual function deterioration (EES: 0% vs. TCS: 44%; *p* = 0.012) among EES compared to TCS. Similarly, Jimenez et al. [54] conducted a meta-analysis comparing the TCS and EES for suprasellar meningiomas. Their analysis showed that EES had significantly higher odds of visual improvement (Odds Ratio [OR] = 3.24, *p* = 0.0053) compared to TCS but had significantly higher odds of a cerebrospinal fluid leak (OR = 3.71, *p* = 0.0098) compared to TCS. Although generally multifactional, our results, in addition to the literature, highlight a better postoperative recovery related to EES and advocate for EES, when possible, to minimize complications attributed to the invasiveness of transcranial access. As a general paradigm, benign tumors within the field of endoscopic endonasal exposure should undergo EES, while malignant tumors in difficult locations may undergo TCS with maximum safe resection to provide the best possible outcomes.

In the realm of skull base surgery, reconstruction techniques and materials are essential aspects of surgical planning. Among available data, 50% of patients had adipofascial anterolateral thigh flap and 37.5% had latissimus dorsi free flap. Several techniques and materials are described in the literature for different locations of the skull base [55,56]. The decision typically relies on different factors including support rigidity and the vascular supply. For instance, since lateral skull base lesions often do not require firm support, the repair of the middle fossa floor is typically performed using a temporalis muscle flap [18]. However, numerous necrosis events have been reported, deeming muscle vasculature maintenance essential and leading others to adopt vascularly rich flaps, such as free rectus abdominis [57,58].

Although only in the univariable analysis, we found that adjuvant radiotherapy and chemotherapy independently decreased the risk of tumor progression. Our results confirmed the established role of adjuvant chemotherapy and radiotherapy in the management paradigm for different skull base lesions. While chemotherapy has been documented to improve survival in malignant lesions, radiotherapy as a primary or adjuvant modality aims mainly to halt tumor progression. Studies that investigated the efficacy of radiotherapy for meningiomas and schwannomas reported high 10-year PFS rates of up to 90–98% [59,60,61,62,63,64,65,66]. However, since different tumor types respond differently to various modalities, it is essential to tailor a personalized management plan based on the patient tumor pathology and their clinical condition.

### 4.3. Limitations

Our results were limited by several factors, including the retrospective nature of the included articles, their inclusion criteria, methodology, treatment protocols, and clinical outcomes evaluated in each included article. Different pathologies were included with insufficient data to delineate the analysis based on the tumor type. The statistical power of multiple endpoints was reduced by the small sample sizes of the present article. Further studies are needed to investigate the impact of different surgical approaches on various pathologies and describe their complication profile and reconstruction methods.

## 5. Conclusions

The ITF is invaded by various neoplastic pathologies, presenting a management challenge due to the wide range of invading tumors that exhibit different biological behaviors. Tumor pathology and overall patient condition should be used to devise a multimodal treatment regimen combining surgical resection, chemotherapy, and/or radiotherapy to optimize therapeutic benefit and minimize postoperative morbidity.

## Figures and Tables

**Table 1:** Demographics and clinical characteristics of the cohort (*n* = 106).

Variable	*n* (%)
Age, years	46 (IQR, 32–55)
Male sex	63 (59.4%)
Tumor size, cm^3^	24.3 (15.2–42)
Involved structures	*n* = 111
Pterygopalatine fossa	29 (26.1%)
Temporomandibular Joint	14 (12.6%)
The orbit	14 (12.6%)
Maxilla	12 (10.8%)
Cavernous sinus	10 (9.0%)
Middle cranial fossa	10 (9.0%)
Nasopharynx	9 (8.1%)
Paranasal sinus	6 (5.4%)
Zygomatic arch	3 (2.7%)
Hard and soft palate	1 (0.9%)
Temporomandibular fossa	1 (0.9%)
Petroclival region	1 (0.9%)
Oropharynx	1 (0.9%)
Histopathology	*n* = 92
Schwannoma	24 (26.1%)
Meningioma	13 (14.1%)
Synovial chondromatosis	11 (12.0%)
Squamous cell carcinoma	5 (5.4%)
Adenoid cystic carcinoma	5 (5.4%)
Embryonal rhabdomyosarcoma	4 (4.4%)
Synovial sarcoma	3 (3.3%)
Lipomas	2 (2.2%)
Solitary fibrous tumor	2 (2.2%)
Pigmented villonodular synovitis	2 (2.2%)
Synovial sarcoma	2 (2.2%)
Neurofibroma	2 (2.2%)
High-grade round-cells	2 (2.2%)
Undifferentiated spindle-cells	2 (2.2%)
Chondrosarcoma	2 (2.2%)
Ameloblastoma	1 (1.1%)
Sclerosing epithelioid fibrosarcoma	1 (1.1%)
Alveolar soft part sarcoma	1 (1.1%)
Hemangiopericytoma	1 (1.1%)
Mucoepidermoid carcinoma	1 (1.1%)
Nasopharyngeal carcinoma	1 (1.1%)
Chondroma	1 (1.1%)
Nasopharyngeal angiofibroma	1 (1.1%)
Capillary hemangioma	1 (1.1%)
Venous malformation ^a^	1 (1.1%)
Leiomyosarcoma	1 (1.1%)
Presentation symptoms	*n* = 119
Facial hypoesthesia	22 (18.5%)
Auricular/preauricular pain	20 (16.8%)
Headaches	11 (9.2%)
Jaw deviation	11 (9.2%)
Hearing loss	9 (7.6%)
Facial pain	8 (6.7%)
Trismus	6 (5.0%)
Temporomandibular Joint pain	4 (3.4%)
Diplopia	3 (2.5%)
Decreased vision	3 (2.5%)
Nasal obstruction/congestion	2 (1.7%)
Vertigo	2 (1.7%)
Trigeminal neuralgia	2 (1.7%)
Exophthalmos	2 (1.7%)
Otitis media	1 (0.8%)
Dysarthria	1 (0.8%)
Dysphagia	1 (0.8%)
Dementia	1 (0.8%)
Ptosis	1 (0.8%)
Involved cranial nerves	*n* = 15
CN III	2 (13.3%)
CN IV	1 (6.7%)
CN V	7 (46.7%)
CN VI	3 (20.0%)
CN VII	1 (6.7%)
CN IX	1 (6.7%)
Patients had surgical resection	97 (95.1%)
Surgical approach	*n* = 95 *
TCS	70 (73.7%)
EES	25 (26.3%)
Mandibulotomy approach	16 (17.8%)
Condylotomy with posterior disc attachment release	14 (15.6%)
Unspecified endoscopic endonasal approach	13 (14.4%)
Middle fossa/zygomatic approach	9 (10.0%)
Endoscopic prelacrimal recess approach	6 (6.7%)
Endoscopic extended medial maxillectomy	5 (5.6%)
Cervical approach	3 (3.3%)
Transmandibular approach	3 (3.3%)
Submandibular and preauricular approach	2 (2.2%)
Latero-facial approach	2 (2.2%)
Degloving approach	2 (2.2%)
Parotidectomy incision	2 (2.2%)
Submandibular cutaneous incision	1 (1.1%)
Preauricular subtemporal approach	1 (1.1%)
Orbito-zygomatic approach	1 (1.1%)
Left temporal craniotomy	1 (1.1%)
Temporal and buccal incision	1 (1.1%)
Weber Fergusson approach	1 (1.1%)
Transzygomatic arch approach	1 (1.1%)
Transcochlear approach	1 (1.1%)
Orbito-zygomatic osteotomy	1 (1.1%)
Zygomatic osteotomy	1 (1.1%)
Antero-lateral, transcraniofacial, subtemporal, extradural approach	1 (1.1%)
Coronal approach	1 (1.1%)
Endoscopic Denker’s approach	1 (1.1%)
Extent of surgical resection	*n* = 84
Gross-total resection	62 (73.8%)
Subtotal resection	17 (20.2%)
Biopsy	5 (6.0%)
Surgical complications	
All complications	35 (33.0%)
Lingual nerve complication	8 (22.9%)
Inferior alveolar nerve complication	7 (20.0%)
Facial paresis	7 (20.0%)
Partial facial numbness	3 (8.6%)
Facial pain	2 (5.7%)
Deep vein thrombosis	2 (5.7%)
CN III Deficit	1 (2.9%)
CN VI Deficit	2 (5.7%)
CN VII Deficit	1 (2.9%)
CN VIIII Deficit	1 (2.9%)
Wound dehiscence	1 (2.9%)
Reconstruction techniques and material	*n* = 8
Adipofascial antero-lateral thigh flap	4 (50%)
Latissimus dorsi free flap	3 (37.5%)
Antero-lateral thigh flap	1 (12.5%)
Adjuvant chemotherapy	*n* = 14 (13.2%)
Methotrexate	1 (7.1%)
Cisplatin	1 (7.1%)
Ifosfamide	1 (7.1%)
Doxorubicin	1 (7.1%)
Unknown	10 (71.4%)
Adjuvant radiotherapy	*n* = 16 (15.1%)
EBRT	3 (18.8%)
GKRS	2 (12.5%)
Proton beam therapy	1 (6.3%)
Unknown	10 (62.5%)
Recurrence	15 (14.2%)
Status	*n* = 97
Alive	88 (83.0%)
Dead	18 (17.0%)
Survival rates	
5-year OS	20%
5-year PFS	61%
Median follow-up time in months (IQR)	28 months (12.3–45.8)

Data are reported as median and interquartile range (Q1–Q3) or frequencies and percentages. CN, cranial nerve; GKRS, Gamma Knife radiosurgery; EBRT, external beam radiotherapy; TCS, transcranial surgery EES, endoscopic endonasal surgery. * The transcranial approaches in the table do not sum up to the total number of transcranial surgery because many surgical approaches were unclarified. ^a^ This was reported by Liu et al. [16].

**Table 2:** Patient outcomes based on surgical approaches and tumor histology.

Surgical Access Based on Tumor Histology	Number of Patients with Available Data	Endonasal Access No. (%)	*p*-Value ^
Trigeminal schwannoma	22	19 (86%)	<0.01
Grade 1 meningioma	12	2 (16.7%)	
Squamous cell carcinoma	3	1 (33%)	
Grade 2 meningioma	1	1 (100%)	
The rest	54	0	
**Complication Based on Surgical Access**	**Total Number of Patients with Each Access**	**Complications** **No. (%)**	***p*-Value ^**
Transcranial access	70	33 (47.1%)	0.01
Endonasal access	25	2 (8.0%)	
**Complication Based on Surgical Approach**	**Total Number of Patients with Each Approach**	**Complications** **No. (%)**	***p*-Value ^**
Parotidectomy incision	2	2 (100.0%)	<0.01
Transzygomatic arch approach	1	1 (100.0%)	
Latero-facial approach	2	2 (100.0%)	
Mandibulotomy approach	16	9 (56.3%)	
Middle fossa/zygomatic approach	9	3 (33.3%)	
Endoscopic prelacrimal recess	6	1 (16.7%)	
Cervical approach	3	1 (33.3%)	
Condylotomy with posterior disc attachment release	14	2 (14.3%)	
Endoscopic endonasal approach	13	1 (7.7%)	
**Recurrence Rate Based on Tumor Histology**	**Total Number of Patients**	**Death** **No. (%)**	***p*-Value ^**
Trigeminal schwannoma	22	1 (5%)	0.04
Grade 1 meningioma	12	3 (25%)	
Adenoid cystic carcinoma	5	2 (40%)	
Squamous cell carcinoma	5	1 (20%)	
Synovial sarcoma	5	3 (60%)	
Alveolar soft part sarcoma	1	1 (100%)	
Sclerosing epithelioid fibrosarcoma	1	1 (100%)	
Grade 2 meningioma	1	1 (100%)	
Capillary hemangioma	1	1 (100%)	
The rest	39	0	
**Mortality Rates Based on Tumor Histology**	**Total Number of Patients**	**Death** **No. (%)**	***p*** **-Value ^**
Trigeminal schwannoma	22	3 (13.6%)	<0.01
Grade 1 meningioma	12	1 (8.3%)	
Adenoid cystic carcinoma	5	1 (20%)	
Synovial sarcoma	5	4 (80%)	
Neurofibroma	2	2 (100%)	
Undifferentiated spindle cells	2	2 (100%)	
Alveolar soft part sarcoma	1	1 (100%)	
Sclerosing epithelioid fibrosarcoma	1	1 (100%)	
The rest	42	0	

^ Chi-square test.

**Table 3:** Overall survival Cox proportional hazards of patient and treatment characteristics.

Variable	Overall Survival			
	Univariable Analysis		Multivariable Analysis	
	HR (95% CI)	*p* Value	HR (95% CI)	*p* Value
Age	1.0 (0.9–1.0)	0.4	1.0 (1.0–1.0)	0.45
Male sex	0.9 (0.5–1.4)	0.6	1.4 (0.6–3.4)	0.42
Schwannoma	0.6 (0.3–1.1)	0.09	NA	NA
Meningioma	1.5 (0.6–3.8)	0.4	NA	NA
Tumor volume cc	1.0 (0.9–1.0)	0.3	1.0 (1.0–1.0)	0.09
EES (Vs. TCS)	0.7 (0.4–1.1)	0.2	NA	NA
GTR (Vs. STR)	1.1 (0.8–1.6)	0.2	6.5 (1.4–29.0)	0.02
Adjuvant chemotherapy	0.6 (0.3–1.2)	0.1	0.6 (0–52.4)	0.82
Adjuvant radiotherapy	0.9 (0.4–1.9)	0.7	NA	NA

**Table 4:** Progression-free survival Cox proportional hazards of patient and treatment.

Variable	Progression-Free Survival			
	Univariable Analysis		Multivariable Analysis	
	HR (95% CI)	*p* Value	HR (95% CI)	*p* Value
Age	0.9 (0.9–1.0)	0.6	1 (0.9–1.2)	0.6
Male sex	0.8 (0.3–2.1)	0.6	9.37 × 10^8^ (0-inf)	1
Schwannoma	0.0 (0-inf)	1	2.2 (0-inf)	1
Meningioma	0.31 (0.1–1.0)	**0.04**	NA	NA
Tumor volume cc	1.0 (1.0–1.0)	0.9	1.0 (1.0–1.0)	0.8
EES (Vs. TCS)	3.7 (0.8–16.4)	**0.08**	2.66 × 10^8^ (0-inf)	1
GTR (Vs. STR)	2.5 (1.5–4.1)	**<0.01**	2.70 × 10^−5^ (0-inf)	1
Adjuvant chemotherapy	0.26 (0.1–0.7)	**<0.01**	9.50 × 10^−11^ (0-inf)	1
Adjuvant radiotherapy	0.32 (0.1–0.9)	**0.02**	NA	NA

**Table 5:** Logistic regression of complications.

Variable	Complications			
	Univariable Analysis		Multivariable Analysis	
	OR (95% CI)	*p* Value	OR (95% CI)	*p* Value
Age > 46 years	2.4 (0.7–9.8)	0.2	5.58 × 10^8^ (1.59 × 10^−209^-NA)	1.0
Male sex	0.3 (0.1–1.1)	0.09	5.58 × 10^8^ (0–1)	0.1
Tumor volume > 24.3 cc	2.1 (0.19–48.4)	0.6	2.6 (0.1–236.5)	0.6
EES (Vs. TCS)	4.1 (1.1–27.1)	0.07	0.4 (0–16.9)	0.6
GTR (Vs. STR)	1.5 (0.8–2.7)	0.18	4.5 (0–1.6 × 10^95^)	1.0

## Supplementary Materials

The following are available online at https://www.mdpi.com/article/10.3390/cancers14215420/s1, Supplementary Table S1: Risk of bias assessments for all included studies. Supplementary Table S2. Overview of included articles.
