# Supplementary material for: Tumors Involving the Infratemporal Fossa: A Systematic Review of Clinical Characteristics and Treatment Outcomes
Source: Cancers (Basel). 2022 Nov 3;14(21):5420. doi: 10.3390/cancers14215420 (PMC9655731; doi:10.3390/cancers14215420)
Supplement: Supplementary file 1 [file cancers-14-05420-s001.zip › Supplementary Table S1.pdf]

Supplementary Table S1. Risk of bias assessments for included studies

**JBI Checklist for Case Series – Criteria**
1. Were there clear criteria for inclusion in the case series?
2. Was the condition measured in a standard, reliable way for all participants included in the case series?
3. Were valid methods used for identification of the condition for all participants included in the case series?
4. Did the case series have consecutive inclusion of participants?
5. Did the case series have complete inclusion of participants?
6. Was there clear reporting of the demographics of the participants in the study?
7. Was there clear reporting of clinical information of the participants?
8. Were the outcomes or follow up results of cases clearly reported?
9. Was there clear reporting of the presenting site(s)/clinic(s) demographic information?
10. Was statistical analysis appropriate?
**Responses Options**: Yes, No, Unclear, Not Applicable (NA)
**Quality Rating**: Poor 0 – 3; Fair 4 – 7; Good 8 – 10

Study	1	2	3	4	5	6	7	8	9	10	Appraisal
Liu et al., 2020 [16]	Yes	Yes	Yes	Yes	Yes	Yes	Yes	Yes	No	Yes	9 – Good
Wang et al., 2019 [9]	Yes	Yes	Yes	Yes	Yes	No	No	Yes	Yes	Yes	8– Good
Shin et al., 2018 [2]	Yes	Yes	Yes	Yes	Yes	Yes	Yes	Yes	No	Yes	9– Good
Lee et al., 2017 [33]	Yes	Yes	Yes	No	Yes	Yes	Yes	Yes	Yes	Yes	9 – Good
Zhou et al., 2016 [1]	Yes	Yes	Yes	Yes	Yes	Yes	Yes	Yes	Yes	Yes	10 - Good
Haidar et al., 2015 [29]	Yes	Yes	Yes	Yes	Yes	Yes	No	Yes	No	Yes	8 – Good
Nomura et al., 2014 [3]	Yes	Yes	Yes	Yes	Yes	Yes	Yes	Yes	No	Yes	9 - Good
Kekatpure et al., 2014 [24]	Yes	Yes	Yes	Yes	Yes	Yes	Yes	Yes	No	Yes	9 - Good
Morace et al., 2012 [22]	Yes	Yes	Yes	Yes	No	Yes	Yes	Yes	Yes	Yes	9 – Good
Zhang et al., 2012 [51]	Yes	Yes	Yes	Yes	Yes	Yes	Yes	Yes	Yes	Yes	10 - Good
Deneuve et al., 2012 [27]	Yes	Yes	Yes	Yes	Yes	Yes	No	Yes	No	Yes	8 – Good
Xu et al., 2011 [26]	Yes	Yes	Yes	Yes	Yes	Yes	Yes	Yes	No	Yes	9 - Good
Al-Daraji et al., 2009 [33]	Yes	Yes	Yes	Yes	Yes	Yes	Yes	Yes	No	Yes	9 - Good
Chatni et al., 2009 [20]	Yes	Yes	Yes	Yes	Yes	Yes	Yes	Yes	No	Yes	9 – Good
Kakudo et al., 2008 [21]	Yes	Yes	Yes	Yes	Yes	Yes	Yes	Yes	No	Yes	9– Good
Folk et al., 2007 [31]	Yes	Yes	Yes	No	Yes	Yes	Yes	Yes	Yes	Yes	9– Good
Galioto et al., 2006 [19]	Yes	Yes	Yes	Yes	Yes	Yes	Yes	Yes	Yes	Yes	10 - Good
Chung et al., 2004 [15]	Yes	Yes	Yes	Yes	Yes	No	Yes	Yes	Yes	Yes	9 – Good
Dare et al., 2003 [30]	Yes	Yes	Yes	Yes	Yes	Yes	No	Yes	No	Yes	8 – Good
Pieper et al., 1999 [35]	Yes	Yes	Yes	Yes	Yes	Yes	Yes	Yes	No	Yes	9 - Good
Krishnamurthy et al., 1998	Yes	Yes	Yes	Yes	Yes	Yes	Yes	Yes	No	Yes	9 - Good

[25]											
Sichel et al., 1994 [28]	Yes	Yes	Yes	Yes	Yes	Yes	Yes	Yes	Yes	Yes	10 - Good
Wenig et al., 1993 [14]	Yes	Yes	Yes	No	Yes	Yes	Yes	Yes	Yes	Yes	9 – Good
Colmenero et al., 1991 [18]	Yes	Yes	Yes	Yes	Yes	Yes	Yes	Yes	No	Yes	9 - Good
Murayama et al., 1988 [6]	Yes	Yes	Yes	Yes	Yes	Yes	No	Yes	Yes	Yes	9 – Good
Shapshay et al., 1976 [7]	Yes	Yes	Yes	Yes	Yes	Yes	No	Yes	No	Yes	8 – Good
Buchanan et al., 1975 [34]	Yes	Yes	Yes	Yes	Yes	Yes	Yes	Yes	No	Yes	9 - Good
Liu et al., 2020 [16]	Yes	Yes	Yes	Yes	Yes	Yes	Yes	Yes	No	Yes	9 - Good
