# Supplementary material for: Tumors Involving the Infratemporal Fossa: A Systematic Review of Clinical Characteristics and Treatment Outcomes
Source: Cancers (Basel). 2022 Nov 3;14(21):5420. doi: 10.3390/cancers14215420 (PMC9655731; doi:10.3390/cancers14215420)
Supplement: Supplementary file 1 [file cancers-14-05420-s001.zip › Supplementary Table S2.pdf]

Supplementary Table S2. Overview of included articles

	Author, Year	Level of evidence	Cohort size (No. of patients)	No of male patients (%)	Age Mean (range)	Surgical approach (No. of patients)	Reconstruction technique and material (No. of patients)	Mean FU in months (range)	Status (No. Patients)
**1**	Liu et al., 2020 [16]	IV	14	8 (57.1)	47.3 (24-73)	Condylotomy with posterior disc attachment release (14)	-	54.8 (36-96)	Alive (14)
**2**	Wang et al., 2019 [9]	IV	1	0 (0)	27	Endoscopic endonasal approach (1)	-	48	Alive (1)
**3**	Shin et al., 2018 [2]	IV	3	0 (0)	52 (38-66)	Endoscopic endonasal approach (3)	-	18 (12-30)	Alive (3)
**4**	Lee et al., 2017 [33]	IV	1	1 (100)	80	Endoscopic Denker's Approach (1)	-	12	Alive (1)
**5**	Zhou et al., 2016 [1]	IV	6	4 (66.7)	42 (29-59)	Endoscopic prelacrimal recess approach (6)	-	26.2 (17-46)	Alive (6)
**6**	Haidar et al., 2015 [29]	IV	2	0 (0)	35.5 (22-49)	Preauricular subtemporal approach (1)/Endoscopic transnasal approach (1)	-	15 (12-18)	Alive (2)
**7**	Nomura et al., 2014 [3]	IV	3	2(66.7)	23.7 (17-27)	Orbito-zygomatic approach (1)/Left temporal craniotomy (1)	Antero-lateral thigh flap (1)	57 (17-108)	Alive (1)/Dead (2)
**8**	Kekatpure et al., 2014 [24]	IV	4	4 (100)	47.8 (33-65)	Mandibulotomy approach (3)	Adipofacial anterolateral thigh flap (4)	-	Alive (4)
**9**	Morace et al., 2012 [22]	IV	1	1 (100)	14	-	-	12	Alive (1)
**10**	Zhang et al., 2012 [51]	IV	8	4 (50)	44.1 (31-61)	Endoscopic endonasal approach (8)	-	30 (10-74)	Alive (8)
**11**	Deneuve et al., 2012 [27]	IV	9	9 (100)	-	Transmandibular (3)/Cervical (3)/Degloving (2)/Coronal (1)	Latissimus dorsi free flap (3)	47.4 (16-96)	Alive (7)/Dead (2)
**12**	Xu et al., 2011 [26]	IV	5	1 (20)	37.8 (21-57)	Endoscopic extended medial maxillectomy (5)	-	33.4 (12-78)	Dead (5)

**13**	Al-Daraji et al., 2009 [33]	IV	2	1 (50)	25 (24-26)	-	-	21 (12-30)	Dead (2)
**14**	Chatni et al., 2009 [20]	IV	13	13 (100)	-	Mandibulotomy approach (13)	-	18.5 (4-49)	Alive (10)/Dead (3)
**15**	Kakudo et al., 2008 [21]	IV	2	2 (100)	67 (53-81)	Temporal and buccal incision (1)/Preauricular-submandibular incision (1)	-	48 (36-60)	Alive (2)
**16**	Folk et al., 2007 [31]	IV	1	1 (100)	19	-	-	15	Dead (1)
**17**	Galioto et al., 2006 [19]	IV	2	1 (50)	44 (24-64)	Submandibular cutaneous incision (1)/Antero-lateral, transcraniofascial, subtemporal, extradural approach (1)	-	2	Alive (2)
**18**	Chung et al., 2004 [15]	IV	1	0	43	-	-	60	Alive (1)
**19**	Dare et al., 2003 [30]	IV	2	2 (100)	18.5 (18-19)	-	-	24	Alive (2)
**20**	Pieper et al., 1999 [35]	IV	9	3 (33.3)	53.9 (32-73)	Middle fossa/zygomatic approach (9)	-	20.7 (2-36)	Alive (8)/Dead (1)
**21**	Krishnamurthy et al., 1998 [25]	IV	2	0 (0)	52 (49-55)	Orbitozygomatic osteotomy (1)/Zygomatic osteotomy (1)	-	3.75 (1.5-6)	Alive (2)
**22**	Sichel et al., 1994 [28]	IV	2	0 (0)	50.5 (47-54)	Trancochlear approach (1)	-	39 (6-72)	Alive (2)
**23**	Wenig et al., 1993 [14]	IV	1	0 (0)	50	-	-	72	Alive (1)
**24**	Colmenero et al., 1991 [18]	IV	2	0 (0)	58 (54-62)	Latero-facial approach (2)	-	18 (6-30)	Alive (1)/Dead (1)
**25**	Murayama et al., 1988 [6]	IV	1	1 (100)	30	Submandibular and preauricular approach (1)	-	29	Alive (1)

**26**	Shapshay et al., 1976 [7]	IV	7	5 (71.4)	57.7 (45-72)	Parotidectomy incision (1)/Transzygomatic arch approach (1)	-	22 (8-36)	-
**27**	Buchanan et al., 1975 [34]	IV	2	0 (0)	23.5 (17-30)	Parotidectomy incision (1)/Weber Fergusson approach (1)	-	10 (8-12)	Alive (1)/Dead (1)

Data are presented as frequency (%) or mean (range), unless otherwise indicated. Values displayed are of included cases only. "-" indicates data were not reported, incalculable, or indistinguishable from a larger cohort. FU, Follow-up.
